# Structural diversity of the coenzyme methylofuran and identification of enzymes for the biosynthesis of its polyglutamate side chain

**DOI:** 10.1016/j.jbc.2021.100682

**Published:** 2021-05-01

**Authors:** Jethro L. Hemmann, Manuel R. Brühwiler, Miriam Bortfeld-Miller, Julia A. Vorholt

**Affiliations:** ETH Zurich, Institute of Microbiology, Zurich, Switzerland

**Keywords:** one-carbon metabolism, bacterial metabolism, biosynthesis, glutamate, mass spectrometry (MS), microbiology, methylotrophy, methylofuran, coenzyme, polyglutamate, Fhc, formyltransferase/hydrolase complex, H_4_MPT, tetrahydromethanopterin, LC-MS, liquid chromatography–mass spectrometry, MFR, methanofuran, MYFR, methylofuran

## Abstract

Methylofuran (MYFR) is a formyl-carrying coenzyme essential for the oxidation of formaldehyde in most methylotrophic bacteria. In *Methylorubrum extorquens*, MYFR contains a large and branched polyglutamate side chain of up to 24 glutamates. These glutamates play an essential role in interfacing the coenzyme with the formyltransferase/hydrolase complex, an enzyme that generates formate. To date, MYFR has not been identified in other methylotrophs, and it is unknown whether its structural features are conserved. Here, we examined nine bacterial strains for the presence and structure of MYFR using high-resolution liquid chromatography–mass spectrometry (LC-MS). Two of the strains produced MYFR as present in *M. extorquens*, while a modified MYFR containing tyramine instead of tyrosine in its core structure was detected in six strains. When *M. extorquens* was grown in the presence of tyramine, the compound was readily incorporated into MYFR, indicating that the biosynthetic enzymes are unable to discriminate tyrosine from tyramine. Using gene deletions in combination with LC-MS analyses, we identified three genes, *orf5*, *orfY*, and *orf17* that are essential for MYFR biosynthesis. Notably, the *orfY* and *orf5* mutants accumulated short MYFR intermediates with only one and two glutamates, respectively, suggesting that these enzymes catalyze glutamate addition. Upon homologous overexpression of *orf5*, a drastic increase in the number of glutamates in MYFR was observed (up to 40 glutamates), further corroborating the function of Orf5 as a glutamate ligase. We thus renamed OrfY and Orf5 to MyfA and MyfB to highlight that these enzymes are specifically involved in MYFR biosynthesis.

The majority of methylotrophic bacteria use a tetrahydromethanopterin(H_4_MPT)-dependent pathway for the oxidation and conversion of the one-carbon unit originating from methanol or methane (1, 2, 3, 4). In addition to the one-carbon carrier H_4_MPT, this pathway requires the presence of a second coenzyme (5), which has recently been structurally elucidated and termed methylofuran (MYFR; Fig. 1*A*) (6). MYFR acts as a formyl carrier and is tightly bound by formyltransferase/hydrolase complex (Fhc) that generates formate from formyl-H_4_MPT (7, 8, 9). H_4_MPT/MYFR-dependent formaldehyde oxidation is closely related to methanogenesis—a type of energy and carbon metabolism found in archaea (10)—and many of the involved enzymes are conserved (1, 5, 11). MYFR is a structural and functional analog of the archaeal coenzyme methanofuran (MFR) (12), with which it shares a similar core structure. In the model methylotroph *Methylorubrum extorquens*, the core structure of MYFR differs from MFR only in the presence of a tyrosine moiety in place of the tyramine residue (6). However, the most important distinction between MYFR and MFR is the large polyglutamate side chain. In the case of MYFR from *M. extorquens*, this polyglutamate chain contains up to 24 glutamate residues, which are both α- and γ-linked and form a branched chain (6, 9). These features are unique to MYFR, as the five known types of archaeal MFR (MFR-a–MFR-e) contain exclusively γ-linked glutamates arranged in a linear chain of a maximum of 12 units (12, 13, 14, 15). The different types of MFR are distinguished by the composition of the side chain, *i.e.*, the number of glutamate residues and the presence of linkers (as in MFR-e) or terminal residues (as in MFR-a/c). At present, MYFR has only been identified in *M. extorquens*, and it is currently unknown if a similar structural diversity exists within bacteria.

**Figure 1:**
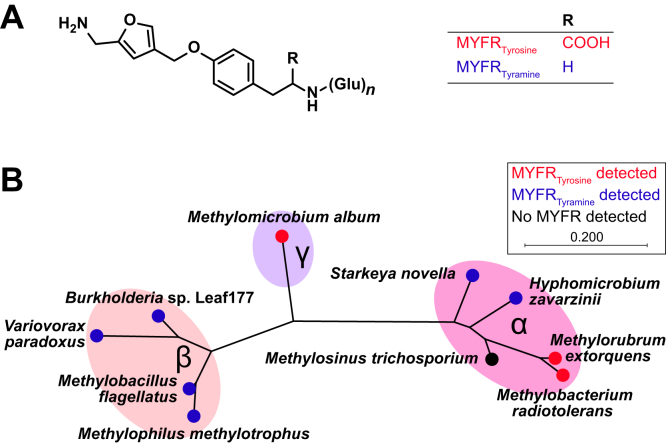
**Structure and phylogenetic distribution of the two types of MYFR identified in this work.***A*, chemical structure of MYFR_Tyrosine_ and MYFR_Tyramine_. The polyglutamate side chain (Glu_*n*_) attached to the core structure consists of a variable number of glutamates (see Fig. 2). *B*, phylogenetic tree based on the 16S rRNA gene of the proteobacterial strains that were screened for the presence of MYFR. All strains harbor genes for the H_4_MPT/MYFR-dependent formaldehyde oxidation pathway. The Greek letters denote the classes Alpha-, Beta-, and Gammaproteobacteria. The *colors of the dots* indicate the type of MYFR that was detected. The phylogenetic tree was calculated using the IQ-TREE web server (48) after alignment of the sequences by Clustal W.

The unique structure of the polyglutamate side chain of MYFR also raises the question of its biosynthesis. In archaea, six enzymes (MfnA–F) have been identified that catalyze the biosynthetic steps required to form the MFR core structure linked to the first γ-glutamate (16, 17, 18, 19). However, the enzymes responsible for the ligation of additional glutamates (all MFRs contain at least two γ-glutamates) as well as the enzymes that introduce the linker or terminal moieties into the side chain of MFR remain to be identified. In *M. extorquens* and other proteobacteria, the proteins Orf22, Orf21, and Orf9 are homologs of the archaeal enzymes MfnB, MfnE, and MfnF respectively. The corresponding bacterial genes are located in close proximity in the genome and are part of the so-called “archaeal-like” gene cluster (5, 20). This cluster contains genes involved in the H_4_MPT/MYFR-linked pathway including *fhcABCD* and genes involved in H_4_MPT biosynthesis. For the transaminase MfnC—responsible for the formation of the amine functionality in the furan moiety (17)—five different homologs are present in *M. extorquens*, and it is unclear which of them fulfills the role of MfnC. As expected and noted before (6), no homolog of MfnA—the l-tyrosine decarboxylase that forms tyramine (16)—is present in *M. extorquens*. Potentially related to that finding is the fact that no homolog of MfnD—the tyramine:l-glutamate ligase involved in the formation of γ-glutamyltyramine (18)—was found either. Analogous to MFR, the enzymes involved in the biosynthesis of the polyglutamate side chain of MYFR have not yet been identified. However, several of the genes in the “archaeal-like” cluster in *M. extorquens* have an unknown function and could therefore play a role in MYFR biosynthesis. Notably, all genes in the cluster are essential for growth on methanol (5, 21). However, prior genetic analysis was complicated by the emergence of secondary mutations and not all mutants could be complemented (21). Further, the identity of MYFR has only been described recently (6), which makes it now possible to directly measure MYFR in mutants of biosynthesis pathways.

Here, we first assessed the structural diversity of MYFR by analyzing different proteobacterial strains by LC-MS to determine whether the structure of MYFR—and consequently its biosynthesis—is likely conserved. We then analyzed mutants of genes from the “archaeal-like” cluster for their ability to biosynthesize MYFR and identified three genes essential for MYFR biosynthesis. Our data suggest that two of these genes, *orfY* and *orf5*, are involved in the elongation of the polyglutamate side chain of MYFR. We thus renamed these genes to *myfA* and *myfB*, respectively, and will use these names throughout the text.

## Results

### Two types of MYFR are present in proteobacteria

To determine the structural diversity of MYFR, we selected several methylotrophic strains (Table S1, Fig. S1). The nine strains covered a diversity of classes within the Alpha-, Beta-, and Gammaproteobacteria (Fig. 1*B*) and included a methylotrophic strain from the *Arabidopsis thaliana* leaf microbiome (*At*-LSPHERE) (22). After cultivation of the strains, MYFR was enriched from cell extracts using anion-exchange solid-phase extraction, assuming that MYFR in these strains would be negatively charged as in *M. extorquens* (6). Using LC-MS, we were able to detect MYFR, as it is present in *M. extorquens*, only in the two strains *Methylobacterium radiotolerans* and *Methylomicrobium album*. For the remaining strains, we then searched the LC-MS data for various derivatives of MYFR. Surprisingly, a series of features corresponded to a decarboxylated form of MYFR (*i.e.*, with masses corresponding to MYFR–CO_2_) in the six strains *Hyphomicrobium zavarzinii*, *Starkeya novella*, *Variovorax paradoxus*, *Burkholderia* sp. Leaf177, *Methylophilus methylotrophus*, and *Methylobacillus flagellatus*. Most likely, such a modification would be localized in the core structure of MYFR. The only available functionality for decarboxylation of the core is the carboxylic acid group that is part of the tyrosine residue (Fig. 1*A*). Indeed, MS/MS fragmentation showed the presence of fragments consistent with a modified core structure containing tyramine instead of tyrosine (Table S2). The core structure of this novel MYFR type is thus identical to all known core structures of MFRs from archaea, which exclusively contain tyramine. To distinguish between the MYFR derivatives, the notation MYFR_Tyrosine_ and MYFR_Tyramine_ will be used (Fig. 1*A*). Notably, all strains tested that belong to the Betaproteobacteria produced MYFR_Tyramine_, while strains with either type of MYFR were found in the Alphaproteobacteria (Fig. 1*B*).

In *Methylosinus trichosporium*, none of the two MYFR derivatives could be detected. To try to identify MYFR with an unknown core structure, we used an untargeted approach to identify features in the LC-MS data that are separated by the mass of one or multiple glutamates from each other. This approach assumed that any unknown MYFR derivatives would be present as a distribution of species with different number of glutamates. Using this method, we identified the polyglutamate-containing compound *p*-aminobenzoyl-Glu_*n*_ with 3 to 5 glutamates in extracts of the bacterium, demonstrating that the approach was working in principle. However, we did not identify a novel MYFR derivative and the coenzyme might therefore be present in concentrations below our detection limit.

**Figure 2:**
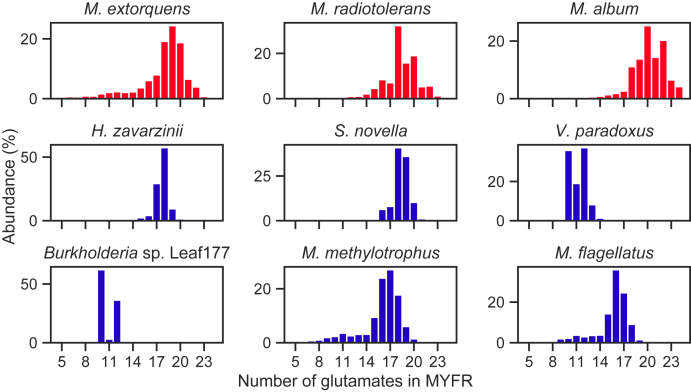
**Distribution of the number of glutamates present in MYFR from the strains analyzed in this work.** The relative abundance of each MYFR species was determined by LC-MS. The *color of the bars* indicates the type of MYFR; *red* for MYFR_Tyrosine_ and *blue* for MYFR_Tyramine_.

For the strains in which MYFR was identified, the number of glutamates attached to the core structure was evaluated (Fig. 2). While most strains showed similar glutamate distributions to the one from *M. extorquens* (most abundant MYFR contained 16–20 glutamates), MYFR from *V. paradoxus* and *Burkholderia* sp. Leaf177 had less than 14 glutamates (most abundant MYFR contained 12 and 10 glutamates respectively). Strikingly, the distribution of MYFR from *Burkholderia* sp. Leaf177 (and to some extent also the one from *V. paradoxus*) showed a distinct gap between MYFR-Glu_10_ and MYFR-Glu_12_, *i.e.*, MYFR-Glu_11_ had a much lower abundance.

### Generation of tyramine and incorporation into MYFR

The discovery of MYFR_Tyramine_ prompted us to investigate the genetic and biosynthetic basis for the incorporation of tyrosine or tyramine into MYFR. In the biosynthesis of MFR, tyramine is generated from tyrosine by MfnA, a pyridoxal phosphate-dependent l-tyrosine decarboxylase first identified in the methanogenic archaeon *Methanocaldococcus jannaschii* (16). To determine whether a similar enzyme is present in bacteria, we used BLAST (23) to search for homologs of MfnA in all strains analyzed above (Table S3). As expected, no homolog of MfnA could be identified for the three strains producing MYFR_Tyrosine_. However, for three of the six strains producing MYFR_Tyramine_, potential *mfnA* genes were identified: Mfla_2033 in *M. flagellatus* (adjacent to the methanol dehydrogenase cluster *mxa*), Snov_0063 in *S. novella*, and VAPA_RS28950 in *V. paradoxus*. All three genes belong to the Pfam protein family PF00282, which contains PLP-dependent decarboxylases. It thus seems likely that these enzymes generate tyramine, which can then be integrated into MYFR. For the remaining three MYFR_Tyramine_-producing strains (*M. methylotrophus*, *H. zavarzinii*, and *Burkholderia* sp. Leaf177), no MfnA homolog was found, suggesting that other tyrosine decarboxylases with weak or no homology to MfnA exist. Alternatively, tyramine might also be produced from a different precursor than tyrosine. An MfnA homolog was also found for *M. trichosporium*, a strain for which we have not been able to identify MYFR so far, thus predicting the presence of MYFR_Tyramine_ in this strain.

**Figure 3:**
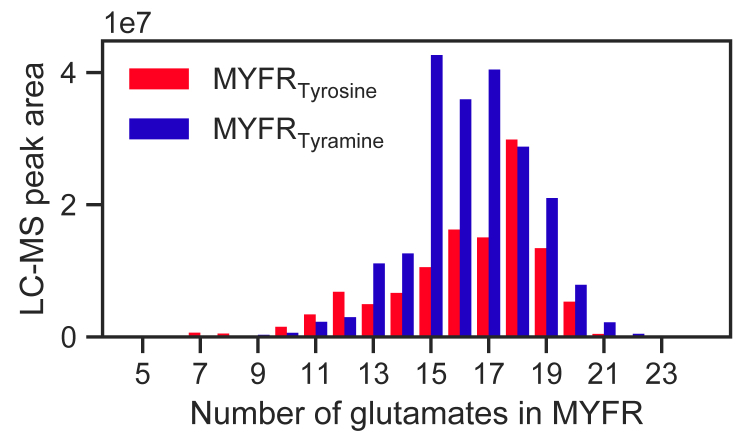
**Distribution of the number of glutamates in MYFR**_**Tyrosine**_**and MYFR**_**Tyramine**_**found in *M. extorquens* grown in the presence of 5 mM tyramine.** MYFR_Tyramine_ constitutes 64% of the total MYFR pool as judged by LC-MS.

In each strain producing MYFR, exclusively one type of MYFR was detected and mixtures of MYFR_Tyrosine_ and MYFR_Tyramine_ were never observed. While tyramine is a specific metabolite that does not seem to be produced in every strain, tyrosine is an indispensable amino acid that must always be present. The incorporation of tyramine into MYFR_Tyramine_ (and also MFR) must therefore be specific and no promiscuity of the responsible enzyme be tolerated. The specificity is consistent with MFR biosynthesis, for which the tyramine:l-glutamate ligase MfnD accepts only tyramine as substrate *in vitro* (18). For strains that do not synthesize tyramine—and consequently are only able to produce MYFR_Tyrosine_—the biosynthetic enzyme might be more relaxed. To test whether the MYFR_Tyrosine_-producing strains would principally be able to incorporate tyramine, we cultivated *M. extorquens* in minimal medium supplemented with 5 mM tyramine and analyzed the cell extract for the presence of the two types of MYFR by LC-MS. Notably, *M. extorquens* was indeed able to take up tyramine and incorporate it into MYFR, as 64% of the total MYFR pool was made up by MYFR_Tyramine_ (Fig. 3). This result demonstrates that the biosynthetic enzyme responsible for tyrosine incorporation is unable to discriminate between tyrosine and tyramine in *M. extorquens*. Likely, this is also the case for the other strains producing MYFR_Tyrosine_.

### The genes *myfA*, *myfB*, and *orf17* are involved in MYFR biosynthesis

Our analysis of MYFR from different methylotrophic strains revealed that its structure is largely conserved, since MYFR in all cases consisted of a core structure (containing either tyrosine or tyramine) and a polyglutamate side chain of at least ten glutamates. The biosynthesis of MYFR is thus likely also conserved, although some of the enzymes must have different specificities to distinguish between tyrosine and tyramine. To identify additional proteins involved in the biosynthesis of MYFR, we focused on the genes with unknown function in the “archaeal-like” gene cluster (5, 21). We analyzed strains with deletions in the three genes *myfB* (*orf5*; in *M. extorquens* AM1), *orf17* (in *M. extorquens* PA1; harboring additionally an *mxaF* deletion, see Experimental procedures), and *myfA* (*orfY*; in *M. flagellatus* KT) to determine whether MYFR biosynthesis is affected by the mutation. As the Δ*myfA* mutant could not be obtained in *M. extorquens* and appears to be lethal, a previously described mutant in *M. flagellatus* was used (20), assuming that the biosynthesis will be similar in both strains. After cultivation of the strains and fractionation of the cell extracts by anion-exchange chromatography, we used LC-MS to detect MYFR. While a WT control contained MYFR as expected (with 6 up to 24 glutamates), MYFR could not be detected in all three mutants. The absence of MYFR in the deletion strains indicates the involvement of *myfA*, *myfB*, and *orf17* in MYFR biosynthesis.

Both MyfA and MyfB are—based on their sequence—predicted to be members of the ATP-grasp superfamily (24, 25), which contains among others ribosomal protein S6 modification enzyme (RimK) (26, 27), d-alanine:d-alanine ligase (28), and glutathione synthetase (28). All members of this family share the unusual ATP-grasp fold for nucleotide binding and most of them catalyze a ligation between a carboxylate and a nucleophile (usually an amine or thiol) *via* an acylphosphate intermediate (24, 25).

Part of the sequence of MyfA shows similarity to MfnD from the methanogen *M. jannaschii*. MyfA might therefore be involved in an analogous step in MYFR biosynthesis and link one or multiple glutamates with tyrosine or tyramine. Interestingly, in addition to MyfA, the strains *M. flagellatus*, *M. album*, *Burkholderia* sp. Leaf177, *V. paradoxus*, *M. methylotrophus*, and *S. novella* contain a second protein that is even more similar to MfnD and thus probably the paralog (Table S4). For *M. flagellatus*, it was shown that deletion of this gene (*orf1*, Mfla_1650) leads to increased sensitivity toward formaldehyde (20).

MyfB (from *M. extorquens*) shows a sequence identity of 33% to MptN (MJ_0620) from *M. jannaschii*. MptN has been characterized as an H_4_MPT:α-l-glutamate ligase (29) and is responsible for the formation of tetrahydrosarcinapterin (H_4_SPT), a derivative of H_4_MPT that is generated through the addition of a glutamate residue to the α-carboxylate of the α-hydroxyglutaric acid moiety (30). Since *M. extorquens* does not produce H_4_STP, but rather a dephosphorylated form of H_4_MPT (dH_4_MPT) lacking the phosphate and α-hydroxyglutaric acid moiety (5), there is no need for an H_4_MPT:α-l-glutamate ligase. MyfB is thus likely involved in the addition of glutamates to MYFR.

A putative function of Orf17 in the biosynthesis of MYFR could not be determined based on its sequence. Orf17 belongs to the “histidine biosynthesis protein” family (Pfam PF00977), which is based on ProFAR isomerase (HisA) and imidazole glycerol phosphate synthase (HisF) (31). However, *M. extorquens* PA1 already contains two adjacent genes—Mext_2551 and Mext_2552—annotated with the functions of HisA and HisF. As there is no obvious need for a potential isomerase (HisA) or cyclase (HisF) in the biosynthesis of MYFR (the formation of the furan ring is catalyzed by MfnB/Orf22 (19)), the function of Orf17 might be unrelated to these activities. *M. jannaschii* possesses with MJ_0703 a homolog of Orf17 (26% sequence identity), which has been suggested to be involved in H_4_MPT biosynthesis (32). However, we were able to detect methenyl-dH_4_MPT in the Δ*orf17 M. extorquens* mutant (Fig. S2), thus invalidating that hypothesis at least in bacteria.

To verify that Orf17 is indeed involved in MYFR biosynthesis, we complemented the mutant by expressing *orf17* from a plasmid. This strain produced MYFR with a WT-like glutamate distribution (Fig. S3), thus confirming that the deletion of *orf17* is responsible for the lack of MYFR in the mutant.

### Accumulation of MYFR biosynthesis intermediates in Δ*myfA* and Δ*myfB*

**Figure 4:**
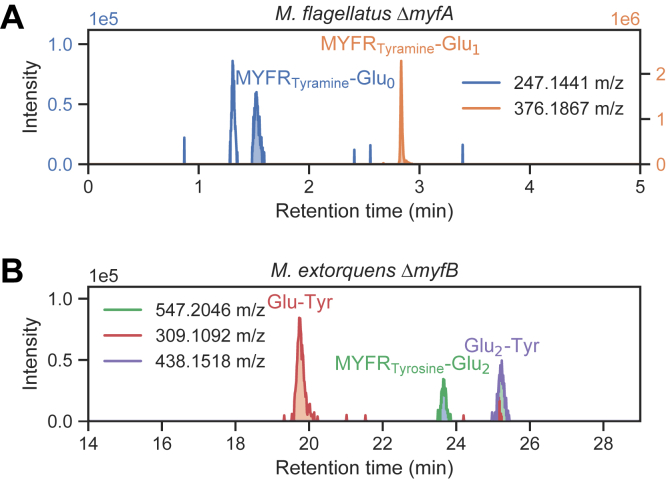
**LC-MS extracted ion chromatograms showing the presence of MYFR biosynthesis intermediates in the deletion strains.** Note that two different LC-MS methods were used in the two panels (see Experimental procedures). *A*, accumulation of MYFR_Tyramine_ biosynthesis intermediates in the Δ*myfA* (=Δ*orfY*) *M. flagellatus* strain. The peaks correspond to the [M + H]^+^ ions of MYFR_Tyramine_-Glu_0_ (MYFR_Tyramine_ core structure only) and MYFR_Tyramine_-Glu_1_. The identity of the peaks was verified by MS/MS, as the typical core structure fragments 213.09 *m*/*z* and 230.12 *m*/*z* were present (see also Table S2). *B*, detection of MYFR_Tyrosine_ biosynthesis intermediates present in the Δ*myfB* (=Δ*orf5*) *M. extorquens* strain. The peaks correspond to the [M − H]^−^ ions of Glu-Tyr, MYFR_Tyrosine_-Glu_2_, and Glu_2_-Tyr. The identity of MYFR_Tyrosine_-Glu_2_ and Glu-Tyr was verified by MS/MS. For further details about the fragmentation of MYFR_Tyrosine_-Glu_2_, see Fig. S4. A mass tolerance of 5 ppm was used to generate all chromatograms.

Even though the deletion strains described above no longer produced MYFR, we expected that intermediates of the interrupted MYFR biosynthesis pathway would continue to be present or even accumulate. We thus analyzed the cell extracts (or purified fractions thereof) by LC-MS for the presence of MYFR intermediates. Surprisingly, in the extract of the Δ*myfA* (=Δ*orfY*) *M. flagellatus* strain, the two intermediates MYFR_Tyramine_-Glu_0_ (MYFR_Tyramine_ without glutamates, *i.e.*, just the core) and MYFR_Tyramine_-Glu_1_ (MYFR_Tyramine_ with one glutamate unit) were accumulating (Fig. 4*A*). The identity of these two compounds was confirmed by MS/MS fragmentation. In a WT cell extract, MYFR_Tyramine_-Glu_0_ was absent while the peak area of MYFR_Tyramine_-Glu_1_ was 39× lower than that in the deletion strain. The accumulation of these two intermediates suggests that MyfA plays a role in the elongation of the polyglutamate side chain, most likely by adding glutamates to the observed intermediates.

To determine biosynthetic intermediates in the Δ*myfB* (=Δ*orf5*) *M. extorquens* mutant, anion-exchange purified fractions of the cell extract were analyzed by LC-MS. In a fraction eluting earlier than the full-length MYFR_Tyrosine_, a peak with a mass corresponding to MYFR_Tyrosine_-Glu_2_ was observed (Fig. 4*B*). MS/MS fragmentation further verified the identity of this compound (Fig. S4). In addition, the same fraction also contained Glu-Tyr and Glu_2_-Tyr (Fig. 4*B*). All three compounds were not observed in a strain that was producing full-length MYFR_Tyrosine_, thus suggesting that these are biosynthesis intermediates that are only present when *myfB* is deleted. The accumulation of the intermediate MYFR_Tyrosine_-Glu_2_ indicates that MyfB acts as a glutamate ligase and extends the two-unit polyglutamate chain present in this intermediate. Similarly as MptN or RimK, MyfA might add glutamates to the C-terminal carboxylate of the precursor. This putative function would be in line with the observation that MYFR in *M. extorquens* has a free amino group at the second glutamate in the chain (9) and hence all further glutamates have to be attached C-terminally. MyfA and MyfB thus likely act sequentially: after MyfA adds a glutamate to the N-terminus of MYFR-Glu_1_, the resulting MYFR-Glu_2_ intermediate can then be elongated C-terminally by MyfB.

In the extract of the Δ*orf17 M. extorquens* mutant, no MYFR-Glu_*n*_ intermediates were observed by LC-MS. Orf17 might thus be involved in an initial step of MYFR biosynthesis, *e.g.*, in the formation of the core structure, which results in intermediates not detectable by our LC-MS method.

### MyfB binds and elongates MYFR *in vivo*

**Figure 5:**
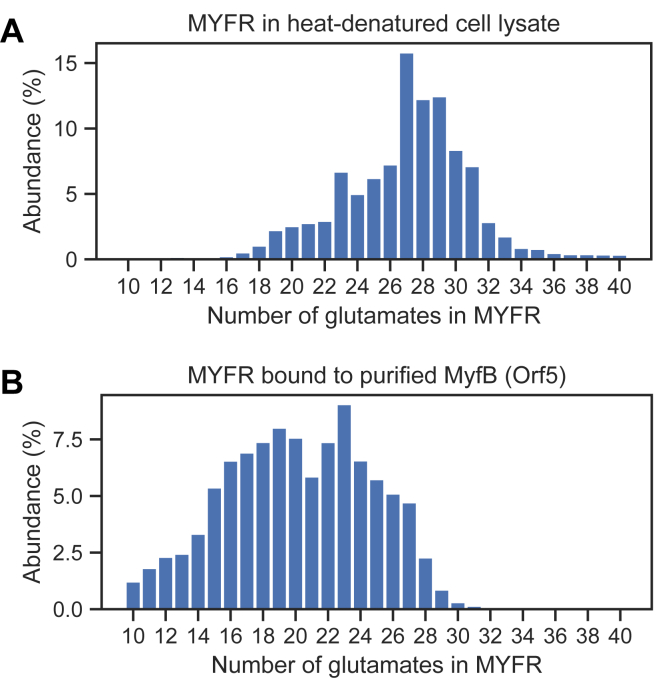
**Distribution of the number of glutamates in MYFR extracted from a *myfB* (o*rf5*) overexpressing *M. extorquens* strain.***A*, glutamate distribution of MYFR extracted from the heat-denatured cell lysate. See Figure 2 for a WT distribution as comparison. *B*, glutamate distribution of MYFR extracted from size-exclusion purified MyfB. Note that both glutamate distributions showed large batch-to-batch variability; however, MYFR from the overexpression strain always contained significantly more glutamates than the WT.

The similarity of MyfB (Orf5) to known glutamate ligases and the accumulation of the intermediate MYFR_Tyrosine_-Glu_2_ in the *M. extorquens* mutant prompted us to further characterize this enzyme. To study its function, we first homologously overexpressed Strep-tagged *myfB* under control of the strong *mxaF* promoter in *M. extorquens*. The cell lysate of this strain was then analyzed by LC-MS to determine whether the overexpression of *myfB* affected the structure of MYFR. Strikingly, MYFR from this strain had a larger polyglutamate side chain and MYFR with up to 40 glutamates was observed (Fig. 5*A*). Further, the distribution of the number of glutamates was broader compared with the WT. The increased expression level of *myfB* thus results in a higher number of glutamates in the chain, confirming that MyfB is a crucial enzyme for the elongation of the polyglutamate side chain.

Next, we partially purified the tagged MyfB from *M. extorquens* by affinity purification and size-exclusion chromatography (Fig. S5, *A* and *B*). To determine whether MyfB was binding MYFR as previously observed for Fhc (9), we heat-denatured a sample of MyfB and analyzed the released MYFR by LC-MS. Surprisingly, purified MyfB had a broad distribution of MYFR bound, containing between 10 and 30 glutamates (Fig. 5*B*). MYFR was still bound to MyfB after two chromatographic steps, indicating a rather strong, yet noncovalent (heat-labile) association.

The glutamate distribution of MYFR in the cell lysate principally corresponds to the sum of the distribution of unbound MYFR and MYFR bound to MyfB and other proteins (such as Fhc). The observation that the MyfB-bound glutamate distribution (Fig. 5*B*) was distinct from the distribution of MYFR in the cell lysate (Fig. 5*A*) suggests that MyfB-bound MYFR represents only a minor subset of the total MYFR pool.

### MyfB catalyzes the synthesis of polyglutamates from l- and d-glutamate *in vitro*

**Figure 6:**
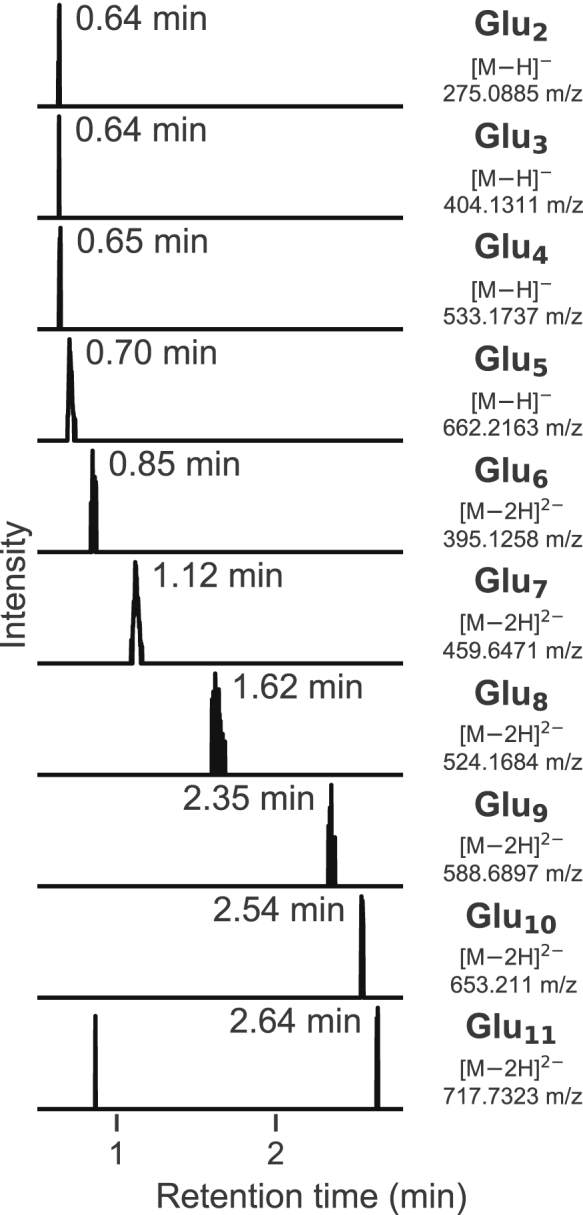
**LC-MS extracted ion chromatograms showing the *in vitro* formation of polyglutamates byMyfB (Orf5).** Polyglutamates with 2 up to 11 units (Glu_2_–Glu_11_) were observed after incubation of MyfB for 23 h with 100 µM of l- and d-glutamate, ATP, and GTP in the presence of 5 mM of MgCl_2_, MnCl_2_, and KCl. A mass tolerance of 5 ppm was used to generate the chromatograms.

To further investigate the function of MyfB (Orf5), we performed *in vitro* assays using purified enzyme produced heterologously in *Escherichia coli* (Fig. S5*C*). Based on the above findings, the putative function of MyfB is to extend the polyglutamate side chain in MYFR, starting from the intermediate MYFR-Glu_2_. However, this intermediate was present only in very low amounts in the Δ*myfB M. extorquens* strain, and we were therefore limited to more available substrates for *in vitro* assays. Assuming that MyfB can form polyglutamates even in the absence of a precursor (similarly as the MyfB-homolog RimK (33)), we performed assays using only glutamate as substrate. When MyfB was incubated with l/d-glutamate and ATP/GTP, indeed the synthesis of polyglutamates with 2 up to 11 units (Glu_2_–Glu_11_) was observed by LC-MS (Fig. 6). Assays performed with either l- or d-glutamate alone also resulted in the synthesis of polyglutamates, demonstrating that both enantiomers can act as a substrate (Fig. S6).

The observed polyglutamate synthesis activity confirms the role of MyfB as a glutamate ligase. However, the amounts of polyglutamates produced were small and required around 1 day of incubation to appear. It is thus likely that the *de novo* formation of polyglutamates is merely a side activity that occurs in the absence of an MYFR precursor. This assumption is consistent with the finding that cell extracts of *M. extorquens* do not contain detectable amounts of polyglutamates.

## Discussion

Our analysis of MYFR from different methylotrophic proteobacteria revealed that there are two main types of MYFR: the previously described MYFR_Tyrosine_ (6) and the herein identified MYFR_Tyramine_ that contains tyramine instead of tyrosine in its core structure. Interestingly, the predominant type of MYFR in the surveyed strains is MYFR_Tyramine_ (six out of nine strains), which has the same core structure as archaeal MFR. Due to the predominance of MYFR_Tyramine_, the missing carboxylic acid group is not expected to significantly affect the function of MYFR. Besides these variances in the core structure, the analysis also highlights differences in the number of glutamates attached to the core (Fig. 2). Notably, MYFR in all strains contained at least ten glutamates, suggesting that this might be the minimal number of units required for MYFR to function as a prosthetic group of Fhc. Based on the crystal structure of Fhc from *M. extorquens* (9), we previously estimated that about five glutamates are necessary to span the mere distance between the MYFR binding site and the two active sites, while a similar number of glutamates might be required to create the branched structure needed for tight binding of MYFR to Fhc. The structure further showed that the branched polyglutamate side chain of MYFR associates with Fhc *via* a large number of electrostatic interactions between the negatively charged glutamates and numerous positive residues of Fhc (9). These amino acids are conserved in methylotrophic bacteria (9), including the ones tested here, indicating a similar binding mode of MYFR in all strains. It thus remains puzzling what purpose the observed structural diversity serves and whether the differences in MYFR also require structural adaptions of Fhc.

Variances in the number of glutamates (both within and between strains) are also observed for the coenzymes tetrahydrofolate (34) and F_420_ (35). The glutamate chains in these coenzymes are catalytically not required and their physiological role is still not entirely clear. However, several functions have been proposed, including increased intracellular retention, enhanced recognition by enzymes, alteration of kinetic parameters, and facilitation of channeling (34, 36). In the case of MYFR, the polyglutamate chain serves a distinct function: not only does it allow tight binding to Fhc, but it also acts as a flexible linker that enables MYFR to reach both active sites of the bifunctional enzyme for the shuttling of formyl units (9).

The structural diversity of MYFR also affects its biosynthesis. In strains producing MYFR_Tyramine_, MfnA is required to decarboxylate tyrosine to tyramine, thus adding a step to the biosynthesis. The selective incorporation of tyramine further requires a biosynthetic enzyme of high specificity to prevent cross-reactivity with tyrosine. In contrast, we showed that the incorporation of tyrosine in MYFR_Tyrosine_-producing strains is nonspecific, as *M. extorquens* was able to synthesize MYFR_Tyramine_ when supplemented with tyramine. At least in *M. extorquens*, the biosynthetic enzymes thus seem to be agnostic to the type of the core structure.

**Figure 7:**
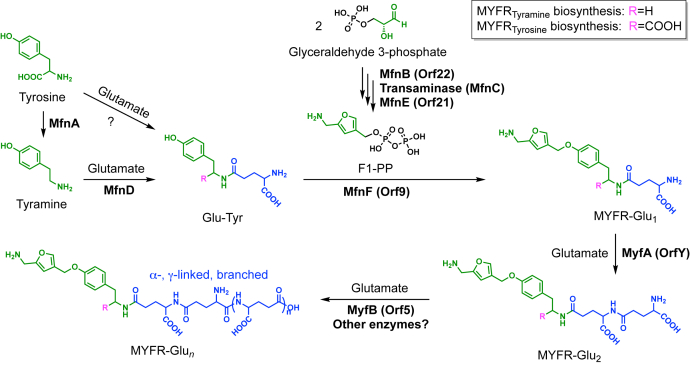
**Proposed biosynthetic pathway for the production of MYFR in bacteria.** The conversion of tyrosine to tyramine only takes place in strains that produce MYFR_Tyramine_ and thus carry the *mfnA* gene. Note that the types of the linkages between the glutamates are unknown; here, they are shown arbitrarily as γ-linkages. F1-PP, 5-(aminomethyl)-3-furanmethanol-pyrophosphate.

Focusing further on the biosynthesis of the polyglutamate side chain of MYFR, we identified OrfY and Orf5 as crucial enzymes for the elongation of the chain. We therefore renamed these proteins to MyfA and MyfB, respectively, highlighting that these are the first enzymes discovered to be specifically involved in the biosynthesis of MYFR. Based on the biosynthetic pathway of archaeal MFR and incorporating the findings of this work, a putative pathway for MYFR biosynthesis is proposed (Fig. 7). The biosynthesis starts with tyrosine, which can either be decarboxylated to tyramine by MfnA (in strains producing MYFR_Tyramine_) or directly be used for the addition of a glutamate unit. The ligation of tyramine with glutamate might be catalyzed by homologs of archaeal MfnD (18), although no homolog was found for the MYFR_Tyramine_-producing strain *H. zavarzinii*. The enzyme catalyzing the ligation of tyrosine with glutamate is still unknown, as most MYFR_Tyrosine_-producing strains do not have a homolog of MfnD (Table S4). The intermediate F1-PP (5-(aminomethyl)-3-furanmethanol-pyrophosphate) is assumed to be formed analogously as in MFR biosynthesis (17, 19) using MfnB (Orf22), a yet to be identified transaminase (MfnC), and MfnE (Orf21). In the next step, MfnF (Orf9) presumably catalyzes the ligation of F1-PP with the glutamate-tyrosine/tyramine dipeptide (Glu-Tyr), resulting in MYFR-Glu_1_. The addition of the second glutamate is catalyzed by MyfA, since MYFR-Glu_1_ is accumulating in the Δ*myfA M. flagellatus* strain. It is still unclear, why additionally MYFR-Glu_0_ (*i.e.*, just the MYFR core) was observed in this strain. This observation also makes it conceivable that tyrosine/tyramine is first ligated with F1-PP and only then the first glutamate is added (potentially still by MfnD). The further elongation of the glutamate chain of MYFR-Glu_2_ is at least partly catalyzed by MyfB, whose deletion in *M. extorquens* leads to the accumulation of MYFR-Glu_2_, while its overexpression results in a drastic increase in the number of glutamates in MYFR (Fig. 5*A*). *In vitro* assays further confirmed its role as a glutamate ligase (Fig. 6). These assays also revealed that MyfB can use both enantiomers of glutamate as a substrate, raising the possibility for the presence of d-glutamate in MYFR. Other, yet to be identified, enzymes might additionally be required for the maturation of the polyglutamate chain, which especially includes the addition of branching sites. The role of Orf17 in MYFR biosynthesis is still unclear and hence not part of the proposed pathway.

In all analyzed strains, MYFR was present as a mixture of species with a varying number of glutamates. Thus, an interesting question concerns the regulation of the length of the polyglutamate chain. The ability of MyfB to noncovalently bind MYFR (Fig. 5*B*) points toward a processive mechanism (37), where the polyglutamate moiety stays bound for multiple rounds of elongation and is only released once its destined length is reached. However, the finding that the overexpression of *myfB* was enough to drastically increase the number of glutamates in MYFR suggests that MyfB does not intrinsically limit the length of the chain, but that the length is rather determined by the expression level of *myfB*. It will thus be interesting to investigate the interaction between MYFR and MyfB and elucidate the mechanism by which the enzyme elongates the chain.

In general, amide bond forming ligases, including ATP-grasp enzymes, are a versatile class of proteins with unique properties for synthetic applications (38, 39, 40). The glutamate ligase activities of MyfA and MyfB thus provide interesting prospects for biocatalysis, *e.g.*, for the production of polyglutamate, a nontoxic biopolymer of industrial relevance (41, 42). Our finding that MyfB is able to synthesize polyglutamates *in vitro* using both l- and d-glutamate demonstrates the potential of this enzyme. Using enzyme engineering, the activity and substrate scope might further be fine-tuned for a given application. Lastly, the identification of the enzymes that introduces the branching into the polyglutamate side chain of MYFR would open up even more potential for biocatalysis, as such enzymes have not yet been described.

## Experimental procedures

### Cultivation of bacterial strains

When possible, the strains used for the MYFR screen (Table S1) were grown in baffled flasks in a minimal medium with 0.5% (v/v) methanol (MMM). MMM is composed of mineral salts (30.3 mM NH_4_Cl, 0.8 mM MgSO_4_), buffer (13.8 mM K_2_HPO_4_, 6.9 mM NaH_2_PO_4_), iron solution (40.3 μM Na_2_EDTA, 10.8 μM FeSO_4_), and trace elements (15.7 μM ZnSO_4_, 12.6 μM CoCl_2_, 5.1 μM MnCl_2_, 16.2 μM H_3_BO_3_, 1.65 μM Na_2_MoO_4_, 1.2 μM CuSO_4_, 20.4 μM CaCl_2_). The strains *V. paradoxus* 351, and *Burkholderia* sp. Leaf177 harbor a lanthanide-dependent methanol dehydrogenase and additionally required LaCl_3_ (60 μM) for growth on methanol. The strain *Burkholderia* sp. Leaf177 did not grow well in liquid minimal medium and was thus cultivated on solid MMM agar plates (1.5% agar). *S. novella* DSM 506 was grown in R2A medium (0.5 g/l yeast extract, 0.5 g/l proteose peptone, 0.5 g/l casamino acids, 0.5 g/l glucose, 0.5 g/l soluble starch, 2.7 mM sodium pyruvate, 1.2 mM K_2_HPOP_4_, 0.2 mM MgSO_4_) supplemented with 0.5% (v/v) methanol.

*M. extorquens* mutants were grown in minimal medium with succinate (MMS), which is identical to MMM except for the replacement of the buffer component with 9.1 mM K_2_HPO_4_ and 11.5 mM NaH_2_PO_4_ and for the addition of 30.8 mM disodium succinate instead of methanol. The *M. flagellatus* mutant was grown in MMM.

All strains were incubated at 28 °C, except for *M. flagellatus* KT, which was grown at 37 °C.

### Extraction and enrichment of MYFR

For the identification of MYFR in the different proteobacterial strains, cells were harvested from liquid cultures or, in case of *Burkholderia* sp. Leaf177, from an agar plate. Cell pellets were extracted three times using 5 ml of 60% methanol in a boiling water bath as described before (6). The cell extracts were purified using weak anion-exchange solid-phase extraction (SPE) columns (Strata X-AW, 33 μm, 30 mg, Phenomenex). After equilibration of the column with two-time 1 ml methanol and two-time 1 ml water, the methanolic extract was applied. The column was washed two times with 1 ml water and 1 ml methanol, before elution with first 1 ml of 0.05% ammonia in 50% methanol and then with 1 ml of 0.25% ammonia in 50% methanol was performed. The first eluate contained mainly compounds with only a few negative charges (less than eight), while the second eluate contained highly negatively charged compounds including MYFR. Eluates were dried *in vacuo* (using a SpeedVac), and the second eluate was usually used for LC-MS analysis (in a few cases, both eluates were combined).

For *M. flagellatus*, the methanolic cell extract was purified slightly differently. After drying *in vacuo* and redissolving with water, the extract was loaded on a strong anion-exchange column (HiTrap Q HP, 1 ml, GE Healthcare) attached to an FPLC system (ÄKTA Purifier, GE Healthcare). A gradient from 200 mM to 2 M ammonium bicarbonate was applied within 15 min (at 1 ml/min) for elution. MYFR eluted at ∼75 mS/cm (∼1.4 M ammonium bicarbonate). The corresponding fractions were dried *in vacuo*, redissolved with water, and heated at ∼80 °C for the removal of the remaining ammonium bicarbonate before they were analyzed by LC-MS. Due to the ability for parallelization and the savings in time, the SPE method was usually preferred for MYFR enrichment.

To determine the presence of MYFR in the deletion strains, the cell pellets were lysed by either passing them three times through a French press cell (Δ*myfB* and Δ*orf17*) or by using boiling methanol/water as described above (Δ*myfA*). In the former case, proteins were removed from the lysate by heat denaturation at 95 °C for 10 min followed by centrifugation. The cell extracts were then purified using strong anion-exchange chromatography as described above. The MYFR_Tyrosine_-Glu_2_ intermediate observed in the Δ*myfB M. extorquens* strain was eluting at ∼38 mS/cm (∼0.8 M ammonium bicarbonate). The MYFR_Tyramine_-Glu_0/1_ intermediates observed in the Δ*myfA M. flagellatus* strain were detected by LC-MS directly in the cell extract.

For the extraction of MYFR from the *myfB* overexpressing *M. extorquens* strain, a small amount of a cell lysate obtained by French press was heated at 99 °C for 15 min to denature proteins. After centrifugation, the sample was diluted with 1 ml of water and purified using SPE as described above.

To extract MYFR bound to purified MyfB, a small protein sample was heat-denatured at 100 °C for 10 min and diluted to 10 ng/μl for LC-MS analysis.

### LC-MS measurements

For the measurement of MYFR, nanoscale ion-pair reversed-phase LC-MS (using tributylamine as ion-pair reagent) was used (43). Measurements were performed on a Q Exactive Plus orbitrap mass spectrometer (Thermo Fisher Scientific) operated in negative mode and coupled to a nano-2D Ultra LC system (Eksigent/AB SCIEX) as described before (9). The cell extracts were diluted to 1 to 10 μg/μl cell dry weight (cdw; assuming ∼300 μg/ml cdw at OD_600_ = 1) with solvent A (230 μM tributylamine, 230 μM acetic acid, and 3% methanol in water, adjusted to pH 9.0 with ammonia) and 1 μl was injected. The low-abundant MYFR_Tyrosine_-Glu_2_ biosynthesis intermediate was also measured using this method.

For the detection of the MYFR_Tyramine_-Glu_0/1_ intermediates and the polyglutamates produced *in vitro*, a less sensitive method was sufficient. Here, the mass spectrometer was coupled with a Dionex UltiMate 3000 UHPLC (Thermo Fisher Scientific). LC separation took place on a Kinetex Polar C18 column (2.6 μm, 100 Å, 2.1 × 100 mm, Phenomenex). Solvent A was 1.1 M formic acid with 0.2 M ammonia, solvent B was acetonitrile, and solvent C was water. The following gradient was applied at 500 μl/min (% A/B/C): 0 min, 5/3/92; 0.5 min, 5/3/92; 3.5 min, 5/50/45; 4 min, 5/95/0; 7 min, 5/95/0; 7 min, 5/3/92; 10 min, 5/3/92. Heated electrospray ionization (HESI) was performed at 380 °C with a spray voltage of 3 kV or −2.5 kV in positive or negative mode respectively. The sheath and aux gas flow rates were set to 60 and 20, respectively, the capillary was heated to 275 °C, and the S-lens RF level was set to 50. The maximum injection time was 50 ms (AGC target 1e6) and spectra were recorded as centroids using a resolution of 35,000 in the range of 120 to 1200 m/z (270–1200 m/z for improved sensitivity). For cell extracts, a volume corresponding to 10 μg cdw was injected. The samples from the *in vitro* assays were analyzed at a concentration of 2 to 4 μM of (initial) substrate and 25 μl were injected.

MS/MS fragmentation was generally performed using the same method as for MS1 detection; however, an additional scan event was added for fragmentation of the precursor. For the fragmentation of MYFR_Tyramine_-Glu_16_ from *M. flagellatus*, the UHPLC LC-method was used instead of the nanoscale ion-pair method.

### LC-MS data analysis

For basic data analysis, Xcalibur Qual Browser (Thermo Fisher Scientific) was used. The untargeted search for unknown MYFR derivatives was implemented as a Python script using eMZed2 (44). After feature finding, mass differences corresponding to one or multiple glutamate units (129.0426 Da) were identified between the (monoisotopic) masses of all features with an annotated charge state. The resulting list was then filtered manually to identify series of features where each feature is separated by one glutamate unit.

The distribution of the number of glutamates in MYFR was determined using eMZed2 as described before (6, 9). Extracted ion chromatograms of the MYFR species with different number of glutamates (core + *n* Glu; core MYFR_Tyrosine_: C_15_H_18_N_2_O_4_, core MYFR_Tyramine_: C_14_H_18_N_2_O_2_, Glu: C_5_H_7_NO_3_) were generated with usually 5 ppm tolerance for the charge states with 1 ≤ *z* ≤ 8 (1 ≤ *z* ≤ 14 for the large MYFRs observed upon *myfB* overexpression) and for all natural isotopologues with at least 9% abundance. The chromatograms were integrated over a given retention time window and the peak areas for the different charge states and isotopologues were summed for each MYFR species. Retention time windows were manually adjusted where necessary.

### Gene deletion mutants

The gene deletion mutants Δ*myfB* (*orf5*, MexAM1_META1p1764) in *M. extorquens* AM1 (21) and Δ*myfA* (*orfY*, Mfla_1659) in *M. flagellatus* KT (20) were a gift from Dr Ludmila Chistoserdova (University of Washington, Seattle, WA, USA).

The deletion of *orf17* (Mext_1835) in *M. extorquens* PA1 was generated by homologous recombination using pK18*mobsacB*, a broad-host-range vector for marker-free allelic exchange (45). Briefly, homologous regions downstream and upstream of *orf17* were amplified by PCR using overlapping primers (Table S5). The homologous regions were fused by overlap PCR and cloned into pK18*mobsacB* between the XbaI and HindIII sites. After transformation into electrocompetent *M. extorquens* PA1 cells, selection for the first crossover event was performed by plating the cells on MMS medium supplemented with kanamycin (50 μg/ml). Colonies were then selected for the second crossover event by plating them on MMS supplemented with 5% (w/w) sucrose. To prevent the picking of sucrose-resistant *sacB* mutants instead of double crossovers, the resulting colonies were plated again on MMS with kanamycin. Colonies unable to grow on that plate were verified by colony PCR and sequencing. For unknown reasons, the deletion of *orf17* could only be obtained in a Δ*mxaF* (Mext_4150) background. However, the additional *mxaF* deletion is not affecting MYFR biosynthesis.

### Complementation of the Δ*orf17 M. extorquens* mutant

To complement the Δ*orf17* mutant, the *orf17* gene (Mext_1835) was amplified from genomic DNA by PCR (using primers Orf17_fwd/Orf17_rev; Table S5). The forward primer further encoded a strong ribosomal binding site. The restriction sites HindIII and BamHI were used to insert the gene into pCM80 (46). The final plasmid was amplified in *E. coli* DH5α and verified by sequencing, before it was transformed into electrocompetent Δ*orf17*Δ*mxaF M. extorquens* PA1 cells. MYFR from the complemented strain was extracted using boiling methanol/water, enriched by solid-phase extraction, and analyzed by LC-MS as described above.

### Cloning of *myfB* for overexpression in *M. extorquens* and *E. coli*

For the tagging and homologous overexpression of *myfB* (*orf5*, Mext_1832) in *M. extorquens* PA1, *myfB* was amplified together with the region 22 bp upstream of the gene (containing the native ribosomal binding site) from genomic DNA by PCR (using primers Mext_orf5_fwd/Mext_orf5_rev; Table S5). The PCR product was restricted with PstI/NcoI, while the vector pCM80 (46) was cut with PstI/BamHI. A C-terminal Strep-tag II (47) was introduced by ordering both strands of the tag sequence as a primer (Strep_1/Strep_2; Table S5) and ligating them together with restricted *myfB* and vector. The final plasmid was amplified in *E. coli* DH5α and verified by sequencing, before it was transformed into electrocompetent *M. extorquens* PA1 cells.

For the heterologous expression of *myfB* in *E. coli*, the gene including the Strep-tag was amplified by PCR from the plasmid described above (using primers Eco_orf5_fwd/Eco_orf5_rev; Table S5). The PCR product and the vector pET-21a(+) (Novagen) were restricted using NdeI and HindIII, followed by ligation. After plasmid amplification in *E. coli* DH5α and verification by sequencing, the final plasmid was transformed into electrocompetent *E. coli* BL21-Gold(DE3) cells (Agilent Technologies).

### Production and purification of MyfB

For the production of Strep-tagged MyfB (Orf5) in *M. extorquens*, transformed cells were grown in MMM medium containing 0.5% to 1% methanol and supplemented with 10 μg/ml tetracycline. The cell pellets were resuspended with 3 ml Tris buffer (25 mM Tris, 300 mM NaCl, pH 7.6) and the cells were lysed by passing them four times through a French press cell. After ultracentrifugation, MyfB was affinity-purified using a StrepTrap HP column (1 ml, GE Healthcare) equilibrated with the buffer used for resuspension. Elution took place using 2.5 mM desthiobiotin in the same buffer. Fractions containing protein were pooled and concentrated in a 10 kDa centrifugal filter (Amicon Ultra, Merck Millipore). SDS-PAGE analysis revealed the presence of two contaminating proteins (Fig. S5*A*), which were identified by in-gel digestion and LC-MS as propionyl-CoA carboxylase (Pcc) A and B. To further purify MyfB, size-exclusion chromatography was performed using a Superose 6 Increase column (24 ml, GE Healthcare) that was run with the same buffer used for affinity purification. PccAB separated from MyfB during that step; however, the protein was still not entirely pure (Fig. S5*B*).

For the production of MyfB in *E. coli*, transformed cells were grown in LB medium supplemented with 100 μg/μl ampicillin. After an OD_600_ of about 0.8 was reached, gene expression was induced by the addition of 0.1 mM IPTG and the culture was switched to 28 °C for overnight (23 h) expression. Cell lysis and StrepTrap purification took place as described above, but using a buffer containing 25 mM Tris, 150 mM NaCl, and 2 mM TCEP at pH 7.8 for resuspension of the pellet and washing of the column and a buffer containing 25 mM Tris, 150 mM NaCl, and 2.5 mM desthiobiotin at pH 7.5 for elution. Concentrated protein was washed once with the resuspension buffer in a 10 kDa centrifugal filter to remove desthiobiotin. SDS-PAGE analysis revealed that MyfA was mostly pure (Fig. S5*C*).

### *In vitro* assays with MyfB

The *in vitro* assays were performed in a total volume of 10 μl and contained 1 μl of 100 μM l- and/or d-glutamate, ATP, and GTP as well as 0.5 μl of 100 mM MgCl_2_, MnCl_2_, and KCl. A volume of 2 μl of 2.8 mg/ml MyfB purified from *E. coli* was added and the remaining volume (3.5 μl or 2.5 μl, depending on the presence of l- and/or d-glutamate) was filled up with Tris buffer (25 mM Tris, 150 mM NaCl, 2 mM TCEP, pH 7.8). As a negative control, the enzyme was replaced with buffer. The assays were incubated at 28 °C and 2 μl-samples were taken after 0 h and ∼1 day and quenched using 50 μl of 60:20:20 acetonitrile:methanol:0.5 M formic acid. The samples were dried *in vacuo*, dissolved with 50 μl of water, and analyzed by LC-MS for the presence of polyglutamates.

## Data availability

All data are available upon request.

## Supporting information

This article contains supporting information. (49)

## Conflict of interest

The authors declare that they have no conflicts of interest with the contents of this article.
