# Supplementary material for: Structural diversity of the coenzyme methylofuran and identification of enzymes for the biosynthesis of its polyglutamate side chain
Source: J Biol Chem. 2021 May 1;296:100682. doi: 10.1016/j.jbc.2021.100682 (PMC8141765; doi:10.1016/j.jbc.2021.100682)
Supplement: Supplemental Figures S1–S6 and Tables S1–S5 [file mmc1.pdf]

Supporting Information

Structural diversity of the coenzyme methylofuran and identification of enzymes for the biosynthesis of its polyglutamate side chain

Jethro L. Hemmann, Manuel R. Brühwiler, Miriam Bortfeld-Miller, Julia A. Vorholt

Contents:

- Tables S1–S5
- Figures S1–S6

Tables

Table S1. Overview of the strains used for the MYFR screen. For description of the media, see Experimental procedures. The class γ abbreviates γ-Proteobacteria. The shading of the rows indicate the type of MYFR that was detected (red: $MYFR_{Tyrosine}$, blue: $MYFR_{Tyramine}$). MDH, methanol dehydrogenase of type Mxa or Xox; sMMO, soluble methane monooxygenase; pMMO, particulate methane monooxygenase; CBB, Calvin-Benson-Bassham; RuMP, ribulose monophosphate.

Strain	Cultivation medium	Detected MYFR	MDH / MMO	C_1 assimilation cycle
**α-Proteobacteria**				
Methylorubrum extorquens PA1	MMM	$MYFR_{Tyrosine}$	Mxa<br>Xox	Serine
Methylobacterium radiotolerans JCM 2831 (DSM 1819)	MMM	$MYFR_{Tyrosine}$	Mxa<br>Xox	Serine
Methylosinus trichosporium OB3b	MMM	No MYFR detected	Mxa<br>Xox<br>sMMO<br>pMMO	Serine
Hyphomicrobium zavarzinii ZV 580 (DSM 10088)	MMM	$MYFR_{Tyramine}$	Mxa<br>Xox	Serine
Starkeya novella DSM 506	R2A+M	$MYFR_{Tyramine}$	Mxa<br>Xox	Serine<br>CBB
**β-Proteobacteria**				
Variovorax paradoxus 351 (DSM 30034)	MMM+La^{3+}	$MYFR_{Tyramine}$	Xox	Serine<br>CBB
Burkholderia sp. Leaf177 (DSM 102584)	MMM+La^{3+} (agar plate)	$MYFR_{Tyramine}$	Xox	Serine<br>CBB
Methylophilus methylotrophus DSM 46235 (DSM 5691)	MMM	$MYFR_{Tyramine}$	Mxa<br>Xox	RuMP
Methylobacillus flagellatus KT (DSM 6875)	MMM	$MYFR_{Tyramine}$	Mxa<br>Xox	RuMP
**γ-Proteobacteria**				
Methylomicrobium album BG8	MMM	$MYFR_{Tyrosine}$	Mxa<br>Xox<br>pMMO	Serine<br>RuMP

Table S2. Observed MS/MS fragments indicating the presence of tyramine instead of tyrosine in the core structure of MYFR$_{Tyramine}$-Glu$_{16}$ from *M. flagellatus*. The $[M+2H]^{2+}$ ion served as the precursor and its total isotope pattern was fragmented; the precursor mass was therefore set in the center of the pattern (1157.16 *m*/*z*) and an isolation window of 2 *m*/*z* was used. The applied normalized collision energy was 25 and 35 (stepped NCE). Only the fragments that are specific for the MYFR$_{Tyramine}$ core-structure are listed.

**Fragment**	**Formula**	**Measured *m*/*z***	**Calculated *m*/*z***	**Difference (ppm)**
Tyramine 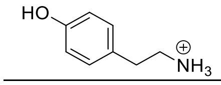	$C_8H_{12}NO^+$	138.0911	138.0913	-1.4
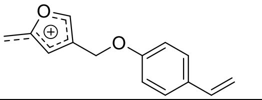	$C_{14}H_{13}O_2^+$	213.0901	213.0910	-4.2
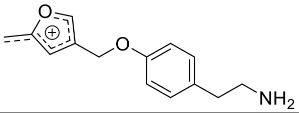	$C_{14}H_{16}NO_2^+$	230.1171	230.1176	-2.2
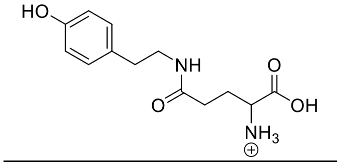	$C_{13}H_{19}N_2O_4^+$	267.1334	267.1339	-1.9
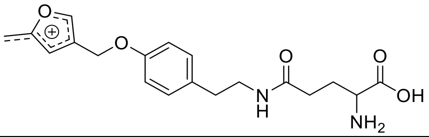	$C_{19}H_{23}N_2O_5^+$	359.1590	359.1602	-3.3

Table S3. Identification of potential MfnA homologs in the selected strains using NCBI BLAST (23). MfnA from *M. jannaschii* (MJ_0050) was used as query sequence. The hit with the lowest E value is listed. Only hits with an E value below 1e-6 were considered homologs and are shown. Default BLAST settings were used, except for the *word size* parameter which was changed to 3 (from 6) in order to increase sensitivity. PF00282 corresponds to the Pfam protein family containing pyridoxal-dependent decarboxylases. The "–" indicates that no significant hit was found.

		Best BLAST hit			
**MYFR type**	**Strain**	**Gene locus tag**	**Annotation**	**Protein family**	**E value**
$MYFR_{Tyrosine}$	*Methylorubrum extorquens*	–	–		–
	Methylomicrobium album	–	–		–
	Methylobacterium radiotolerans	–	–		–
$MYFR_{Tyramine}$	*Methylobacillus flagellatus* KT	Mfla_2033	Pyridoxal-dependent decarboxylase	PF00282	4e-10
	Starkeya novella DSM 506	Snov_0063	Pyridoxal-dependent decarboxylase	PF00282	5e-08
	Variovorax paradoxus B4[a]	VAPA_RS28950	Aspartate aminotransferase family protein	PF00282	3e-33
	Methylophilus methylotrophus	–	–		–
	Hyphomicrobium zavarzinii	–	–		–
	Burkholderia sp. Leaf177	–	–		–
Unknown	*Methylosinus trichosporium* OB3b	CQW49_12090	Cytochrome D ubiquinol oxidase subunit I	PF00282	2e-15

[a]The genome of *Variovorax paradoxus* 351 was not available on NCBI, therefore the strain B4 was used.

Table S4. Identification of potential MfnD homologs in the selected strains using NCBI BLAST (23). Due to the similarity of MfnD and MyfA (OrfY), the corresponding locus tags of MyfA are also shown. MfnD from *M. jannaschii* (MJ_0815) was used as query sequence. The hit with the lowest E value is listed and only hits with an E value below 1e-6 were considered homologs and are shown. Default BLAST settings were used, except for the *word size* parameter which was changed to 3 (from 6) in order to increase sensitivity. The "–" indicates that no significant hit was found.

**MYFR type**	**Strain**	**MfnD**	**MyfA (OrfY)**
$MYFR_{Tyrosine}$	*Methylorubrum extorquens* PA1	-	Mext_1830
	Methylomicrobium album BG8	Metal_3519	Metal_3525
	Methylobacterium radiotolerans JCM 2831	-	Mrad2831_0529
$MYFR_{Tyramine}$	*Methylobacillus flagellatus* KT	Mfla_1650	Mfla_1659
	Starkeya novella DSM 506	Snov_0746	Snov_0751
	Variovorax paradoxus S110[a]	Vapar_3069	Vapar_3083
	Methylophilus methylotrophus DSM 46235	F463_RS0101640	F463_RS0100930
	Hyphomicrobium zavarzinii ATCC 27496[b]	-	F812_RS0112230
	Burkholderia sp. Leaf177	ASG35_15705	ASG35_15815
Unknown	*Methylosinus trichosporium* OB3b	-	-

[a]The genome of *Variovorax paradoxus* 351 was not available on NCBI, therefore the strain S110 was used.
[b]The genome of *Hyphomicrobium zavarzinii* ZV 580 was not available on NCBI, therefore the strain ATCC 27496 was used.

Table S5. List of primers used in this work. Restriction site sequences are underlined.

Name	Function	Sequence
**Deletion of *orf17* in *M. extorquens* PA1**		
KO_Orf17_down_fwd	PCR amplification of homologous region upstream of *orf17*, addition of XbaI site	TAT ATC TAG ACC AGG GAG AGA CCC CGA ATG GC
KO_Orf17_down_rev	PCR amplification of homologous region upstream of *orf17*	GAT GTC TCA CCA TGA CGG GCG GCT GCG CTG
KO_Orf17_up_fwd	PCR amplification of homologous region downstream of *orf17*	GCC CGT CAT GGT GAG ACA TCG CGT GAA CCG
KO_Orf17_up_rev	PCR amplification of homologous region downstream of *orf17*, addition of HindIII site	ATA TAA GCT TCA AGT CGG CGC TGT ATT GCT G
**Complementation of the Δ*orf17* Δ*mxaF* *M. extorquens* mutant**		
Orf17_fwd	PCR amplification of *orf17*, addition of **RBS** and HindIII site	CCC AAG CTT AAG **AAG GAG** ATA TAC CAT GTC TCA CGG TTC TGA GGG G
Orf17_rev	PCR amplification of *orf17*, addition of Strep-tag and BamHI site	AAA GGA TCC TTA TTT TTC GAA CTG CGG GTG GCT CCA AGC GCT GCG CAG CCG CCC GTC ATG
**Overexpression of *orf5*-Strep in *M. extorquens* PA1**		
Mext_orf5_fwd	PCR amplification of *orf5*, addition of PstI site	GGG CTG CAG GAA AAA AAC GGA AAA AGA GCG AAT G
Mext_orf5_rev	PCR amplification of *orf5*, addition of NcoI site, removal of stop codon	AAA CCA TGG CCG AGC GCC GAG ACC AG
Strep_1	Strand 1 of the Strep-tag II (containing NcoI overhang)	CAT GGC AGC GCT TGG AGC CAC CCG CAG TTC GAA AAA TAA G
Strep_2	Strand 2 of the Strep-tag II (containing BamHI overhang)	GAT CCT TAT TTT TCG AAC TGC GGG TGG CTC CAA GCG CTGC
**Expression of *orf5*-Strep in *E. coli* BL21-Gold(DE3)**		
Eco_orf5_fwd	PCR amplification of *orf5*, addition of NdeI site	AAA CAT ATG CGC ATC GGG CTC GCG
Eco_orf5_rev	PCR amplification of *orf5*, addition of HindIII site	CGG AAG CTT TTA TTT TTC GAA CTG CGG GTG GCT C

Figures

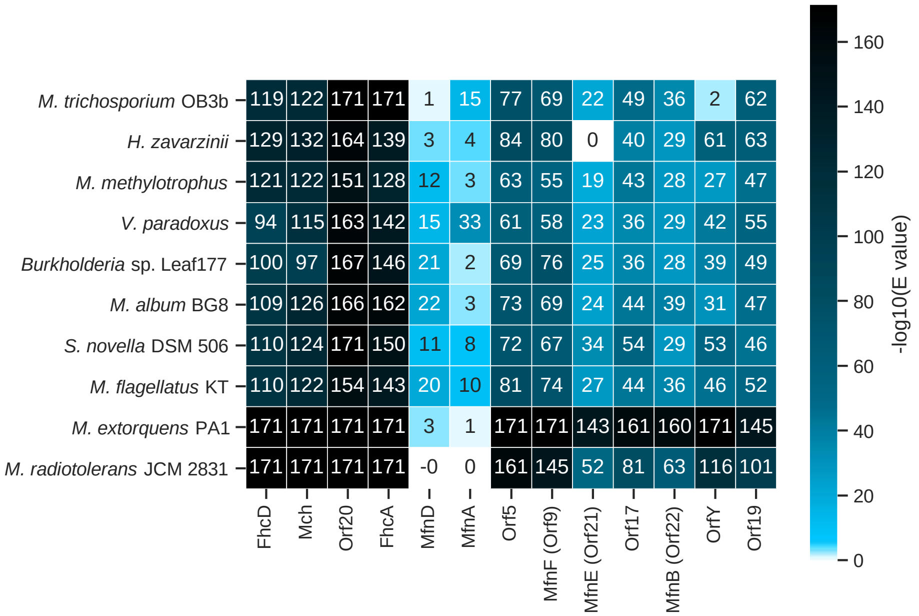

Figure S1. Heatmap showing the presence/absence of different H_4MPT/MYFR-related proteins in the strains used in this work. The values indicate the negative log10 of the E value of the best BLAST hit for each query protein. The larger the value in the heatmap (i.e. the lower the E value), the better the quality of the hit. A value below 6 (E value > 10^{-6}) was interpreted as the absence of the protein in the given strain. E values of 0 were set to the minimal non-zero value in the data set (10^{-171}) to allow log transformation. Query protein sequences were all from *M. extorquens* PA1, except for MfnA and MfnD which were from *M. jannaschii* DSM 2661. The analysis was implemented in Python using the Biopython package (49) to perform online NCBI BLAST searches (23) against the "non-redundant protein sequences" database and using an *Entrez* query for taxonomic filtering.

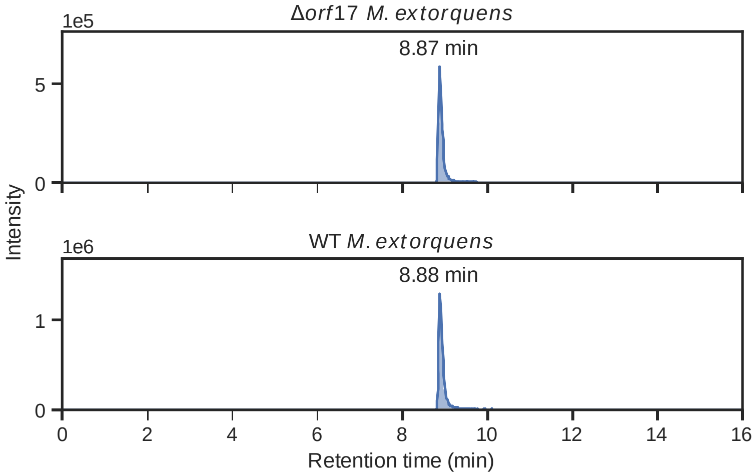

Figure S2. LC-MS extracted ion chromatograms showing the presence of dH_4MPT in the Δ*orf17 M. extorquens* mutant (top) and in the WT control (bottom). The peaks corresponds to the $[M+H]^+$ ion of methenyl-dH_4MPT (577.2617 *m*/*z*). A mass tolerance of 7 ppm was used to generate the chromatograms. Both strains were grown on minimal medium with succinate (MMS) and cell pellets were extracted with 60% boiling methanol as described for the extraction of MYFR. The cell extracts were measured by nanoscale reversed-phase LC-MS using a NanoLC 400 system (Eksigent/AB SCIEX) coupled to an LTQ Orbitrap (Thermo Fisher Scientific). Chromatographic separation was performed at 400 nL/min using a C18 column (Reprosil-Gold 120 C18 3 µm, 0.1 × 100 mm, Dr. Maisch GmbH) with 0.1% formic acid as solvent A and methanol as solvent B. The following gradient was applied: 0 min, 5% B; 2 min, 5% B; 17 min, 90% B; 27 min, 90% B; 28 min, 5% B; 35 min, 5% B. Nanospray ionization settings were: source voltage, 1.9 kV; capillary temperature, 150 °C; capillary voltage, 28 V; tube lens, 100 V. The mass spectrometer was operated in positive FTMS mode with a resolution of 30’000. The amount of injected extract corresponded to 200 ng cdw.

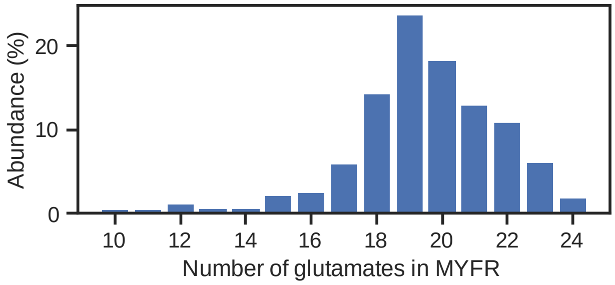

Figure S3. WT-like distribution of the number of glutamates in MYFR extracted from the Δ*orf17*Δ*mxaF* *M. extorquens* mutant upon complementation of the *orf17* deletion.

A

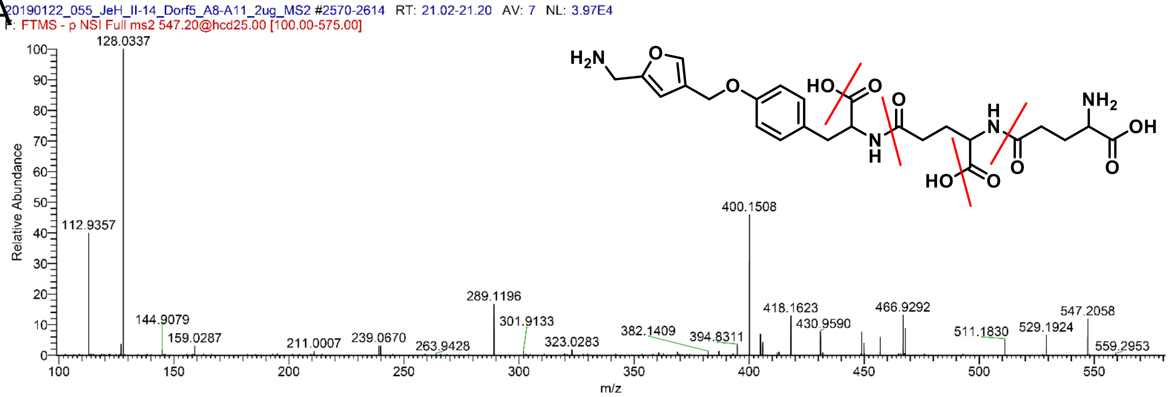

B

Fragment	Formula (neutral)	Measured *m*/*z*	Calculated *m*/*z*	Difference (ppm)
MYFR$_{Tyrosine}$-Glu$_2$ (precursor)	$C_{25}H_{32}N_4O_{10}$	547.2058	547.2046	2.2
MYFR$_{Tyrosine}$-Glu$_2$ – H_2O	$C_{25}H_{30}N_4O_9$	529.1924	529.1940	-3.0
MYFR$_{Tyrosine}$-Glu$_2$ – 2 H_2O	$C_{25}H_{28}N_4O_8$	511.1830	511.1834	-0.8
MYFR$_{Tyrosine}$-Glu$_1$	$C_{20}H_{25}N_3O_7$	418.1623	418.1620	0.7
MYFR$_{Tyrosine}$-Glu$_1$ – H_2O	$C_{20}H_{23}N_3O_6$	400.1508	400.1514	-1.5
MYFR$_{Tyrosine}$-Glu$_1$ – 2 H_2O	$C_{20}H_{21}N_3O_5$	382.1409	382.1408	0.3
MYFR$_{Tyrosine}$-Glu$_0$	$C_{15}H_{18}N_2O_4$	289.1196	289.1194	0.7
Glutamate – H_2O	$C_5H_7NO_3$	128.0337	128.0353	-12.5

Figure S4. MS/MS fragmentation of the $[M–H]^-$ ion of MYFR$_{Tyrosine}$-Glu$_2$ (547.2046 *m*/*z*) that was accumulating in the Δ*myfB* (=Δ*orf5*) *M. extorquens* strain. **(A)** Fragmentation spectrum obtained using a normalized collision energy of 25. The structure indicates the bonds for which dissociation was observed. **(B)** List of the observed fragments. Similar fragments were obtained for full-length MYFR$_{Tyrosine}$ from the WT strain (6).

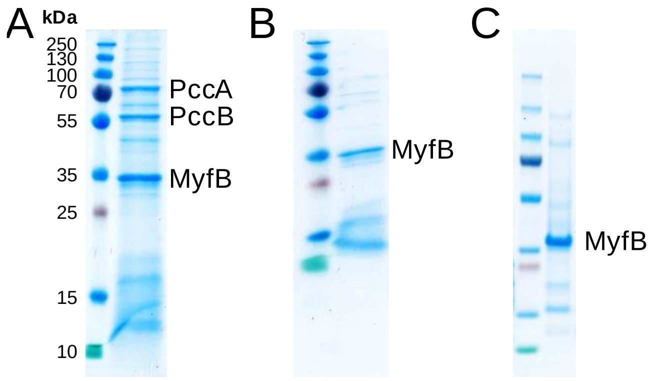

Figure S5. SDS-PAGE of partially purified MyfB (Orf5). The same molecular weight marker is used in both gels and thus only labeled once. **(A)** MyfB homologously produced in *M. extorquens* PA1 after affinity purification using a StrepTrap column. The two upper bands were identified (by in-gel digestion and LC-MS) as the two contaminating proteins propionyl-CoA carboxylase (Pcc) A and B. **(B)** MyfB after further purification by size-exclusion chromatography using a Superose 6 Increase column. **(C)** MyfB heterologously produced in *Escherichia coli* BL21-Gold(DE3) after affinity purification using a StrepTrap column.

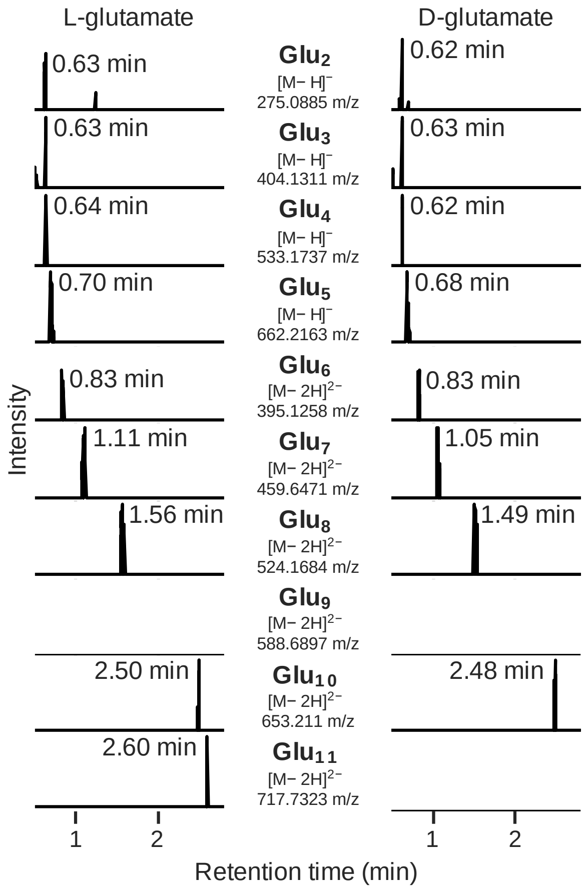

Figure S6. LC-MS extracted ion chromatograms showing the formation of polyglutamates (Glu_n) upon incubation of MyfB (Orf5) for 20 h with 100 µM of L-glutamate (left column) or D-glutamate (right column). The assay additionally contained 100 µM of ATP and GTP and 5 mM of $MgCl_2$, $MnCl_2$, and KCl. Glu_9 was not observed in both assays, while Glu_{11} was only found in the assay with L-glutamate. It is assumed that these species were below the detection limit. A mass tolerance of 5 ppm was used to generate the chromatograms.
